# The prognostic impact of PD-L1 and CD8 expression in anal cancer patients treated with chemoradiotherapy

**DOI:** 10.3389/fonc.2022.1000263

**Published:** 2022-10-07

**Authors:** Angela MY. Chan, Gloria Roldan Urgoiti, Will Jiang, Sandra Lee, Elizabeth Kornaga, Peter Mathen, Rosanna Yeung, Emeka K. Enwere, Alan Box, Mie Konno, Martin Koebel, Kurian Joseph, Corinne M. Doll

**Affiliations:** ^1^ Precision Oncology Hub, Tom Baker Cancer Centre, Department of Oncology, University of Calgary, Calgary, AB, Canada; ^2^ Department of Oncology, University of Calgary, Calgary, AB, Canada; ^3^ Division of Radiation Oncology, University of British Columbia, Vancouver, BC, Canada; ^4^ Department of Pathology and Laboratory Medicine, University of Calgary, Calgary, AB, Canada; ^5^ Radiation Oncology Branch, Center for Cancer Research, National Cancer Institute, National Institutes of Health, Bethesda, MD, United States; ^6^ Department of Radiation Oncology, Evergreen Health, Kirkland, WA, United States; ^7^ Department of Pathology and Laboratory Medicine, University of Saskatchewan, Regina, SK, Canada; ^8^ Division of Radiation Oncology, Department of Oncology, University of Alberta, Edmonton, AB, Canada

**Keywords:** anal cancer, PD-L1, CD8, chemoradiotherapy, immunotherapy

## Abstract

**Background:**

Programmed death-ligand 1 (PD-L1) expression has been shown to be prognostic in many cancer types and used in consideration of checkpoint inhibitor immunotherapy. However, there are very limited and conflicting data on the prognostic impact of PD-L1 in patients with anal squamous cell carcinoma (ASCC). The objectives of this study were to measure the expression of PD-L1 and CD8 in patients with ASCC treated with radical chemoradiotherapy (CRT) and to correlate tumor expression with progression-free survival (PFS) and overall survival (OS).

**Methods:**

Ninety-nine patients with ASCC treated with primary CRT at two tertiary care cancer centers between 2000 and 2013, with available pre-treatment tumors, were included. Tissue microarrays (TMAs) from pre-treatment tumor specimens were stained for PD-L1 and CD8. PD-L1 expression in the tumor and stroma was quantified using HALO image analysis software, and results were interpreted using quantitative methods. The density of CD8 cells within the tumor was interpreted by a trained pathologist semi-quantitatively, using a 0-4 scoring system. Kaplan-Meier analysis with log-rank was used to determine the significance in the association of tumor markers with PFS and OS. Cox multivariate analysis was used to explore independent predictors of PFS and OS.

**Results:**

Of the 99 patients, 63 (64%) had sufficient tumor samples available for full analysis. CD8 high status was documented in 32 of 63 (50.8%) % of cases. PD-L1 expression was positive in 88.9% of cases. Approximately half the patients had tumor PD-L1 ≥ 5%. Patients with tumor PD-L1 ≥ 5% had better OS vs those with lower expression, HR=0.32 (95% CI 0.11-0.87), p=0.027; 10 years OS: 84% for tumor PD-L1 ≥ 5% vs 49% for PD-L1 < 5%. PD-L1 expression was not associated with PFS. On multivariate analysis, tumor PD-L1 ≥ 5% showed a trend to statistical significance for better OS, HR=0.55 (95% CI 0.12- 1.00), p=0.052.

**Conclusions:**

Tumor PD-L1≥5% is associated with OS in patients with ASCC treated with CRT. PD-L1 expression status using this unique cut-point warrants further validation for prognostication in patients with this disease. Future studies are required to determine the benefit of alternative treatment strategies based on PD-L1 status.

## Introduction

Anal cancer, although representing <1% of new cancer diagnoses and <3% of gastrointestinal malignancies, has been increasing in incidence (1). The vast majority of anal cancers are anal squamous cell carcinoma (ASCC) with 1-2 cases per 100,000 per year worldwide (2). The development of ASCC is strongly associated with high-risk human papillomavirus (HPV), which has led to the increase of ASCC incidence in recent years (3, 4). The standard-of-care treatment for patients with pelvic-confined disease is radical chemoradiotherapy (CRT), using a combination of 5-fluorouracil and mitomycin C, and external beam radiotherapy. Although this treatment is generally curative, a subset of patients suffers local and/or distant relapses with salvage or systemic treatments showing suboptimal results (5). The identification of patients who may require alternative treatment approaches is required to improve outcome of patients with this disease, and therefore investigation of novel biomarkers to aid in clinical decision making and in the development of better therapeutic strategies is urgently needed.

Immunotherapy strategies, notably checkpoint inhibitors, have been recently investigated in patients with squamous cell cancers, including patients with anal cancer. Specifically in anal cancer, immunotherapies that influence PD-L1/Programmed cell death protein 1 (PD-1) have been examined in several clinical trials (6–11). The goal is to target the PD-L1/PD-1 interaction by blocking the function of either protein, allowing for T-cell activation and destruction of the tumor. The expression of PD-L1 in tumor and surrounding immune cells was examined to identify patients who might benefit from this immuno-modulating treatment. Using the tumor proportion score (TPS) ≥ 1% as the cut off to select patients who may benefit from pembrolizumab, the combined results of KEYNOTE-028 and KEYNOTE-158 clinical trials showed 73% of patients were PD-L1 positive with an overall response rate of 10.9% (6, 8). These results suggest a ≥ 1% positivity cut-point of PD-L1 expression in anal cancer may not be optimal to select patients who may benefit from the immunotherapy. Aligning with findings in other tumor types, the PD-L1 positivity cut point to achieve clinical benefit is indeed tumor-dependent; for example, non-small cell lung cancer utilizes a TPS ≥ 1%, whereas in advanced triple-negative breast cancer clinical benefit is demonstrated for patients with a combined positive score (CPS) ≥ 10.

While PD-L1 expression is a known target for immune checkpoint blockade, the role of the tumor microenvironment plays a significant role in the effectiveness of cancer immunotherapy. T-cell infiltration into the tumor microenvironment is required for effective tumor killing, including CD8+ cytotoxic T cells. There is interest in studying the tumor microenvironment in HPV-associated cancers, where CD8+ T cell response and enhanced immunoreactivity to viral-associated antigens has been linked to improved survival (12). Furthermore, the association of PD-L1 with HPV status may provide insight into the possibility of combination therapy, including immunotherapy agents. However, there are limited data on the impact of pre-treatment tumor immune status on the outcome of patients with anal cancer undergoing definitive CRT.

In an effort to better understand the molecular environment of anal cancer, a number of studies have tested anal cancers for immune markers (13–19). Many of these studies are small with heterogenous patient and treatment populations, utilize non-quantitative immunohistochemistry (IHC) techniques, and are conflicting in their results. The contradictory results for the association between PD-L1 and OS in anal cancer suggests further investigation is warranted to better understand the impact of this immune biomarker on outcome, as well as its potential to guide therapeutic intervention.

The objectives of this study were to measure the expression of PD-L1 and CD8 in patients with anal cancer treated with CRT, to correlate tumor PD-L1 and CD8 expression with OS and PFS, and to define a PD-L1 positivity cut-point best associated with OS using a highly accurate quantitative image analysis approach. We also aimed to measure the density of tumor CD8+ T cells and correlate PD-L1 and T-cell density with clinical outcomes. Using p16 as an HPV surrogate marker, we additionally examined the association between PD-L1 with p16 and p53 status.

## Materials and methods

### Patients and treatment characteristics

This retrospective study included patients with anal cancer treated with curative-intent CRT between 2000 and 2013 at two major Canadian cancer centers (Tom Baker Cancer Centre, Calgary, AB, Canada and Cross Cancer Institute, Edmonton, AB, Canada). Patients with anal margin cancer and non-invasive anal disease were excluded. Ninety-nine patients with available pre-treatment tumor specimen were included. Clinical and pathologic data were retrieved from the electronic and/or paper charts.

Standard pre-treatment evaluation included physical examination, computed tomography (CT) of the abdomen and pelvis, tumor biopsy, and bloodwork (complete blood count (CBC), electrolytes, liver and renal function tests). HIV testing prior to treatment was performed at the discretion of the treating physician, and not performed in every patient. In addition, patients had weekly bloodwork during treatment, including CBC, electrolytes, urea, and creatinine. Radiotherapy was performed using either 3D conformal or intensity-modulated techniques, with a median dose of 54 Gy in 30 fractions. Chemotherapy consisted of infusional 5-FU during weeks 1 and 5 of RT, with mitomycin C given as IV bolus day 1 of the first or both cycles. After completing treatment, patients were generally followed clinically every three months for the first year, every four months for year two, and every six months to year five. Follow-up imaging was ordered as clinically indicated. Approval for this study was obtained from the University of Calgary Conjoint Health Research Ethics Board.

### Laboratory methods

Pre-treatment formalin-fixed paraffin-embedded (FFPE) tumor samples from patient biopsies were reviewed by the study pathologists (SL and AB) to confirm histology and the adequacy of specimen and location of the tumor for sampling.

### Inducible cell line control

Inducible cell lines were created as previously described (20). pEF1a-rtTA-IRES-GFP from a Tet-ON inducible lentiviral vector system (Takara Bio Group, Mountain View, California, USA) was packaged into lentiviral particles by co-transfecting HEK293FT cells (Thermofisher, Waltham, MA) with psPAX2 and pMD2.G (gifts from Didier Trono (Addgene plasmid numbers 12260 and 12259, respectively)). Viral particles were concentrated from cell culture supernatant and underlaid with 2 mL 20% sucrose in PBS by ultracentrifugation at 50,000 x g for 2 h. The titers were determined by a qPCR lentivirus titration kit and were generally ~108 IU/mL (Applied Biological Materials, Richmond, BC). The pEF1a-rtTA-IRES-GFP will simultaneously produce tetracycline activator and GFP transcription bicistronically.

Lentivirus was used to stably transduce K562 cells (ATCC, CCL-243, Old Town Manassas, Virginia, USA) with the PD-L1 gene under the Tet-On system allowing inducible expression of PD-L1 by addition of varying amounts of Doxycycline. With the addition of Doxycycline, the TRE3G promoter driving PD-L1 expression packaged on a second lentivirus will then respond to the Doxycycline bound Tet activator to induce expression of PD-L1 and mCherry. Hence, cells with successful transduction of both lentiviruses will show both GFP and mCherry expression and appear yellow under a fluorescent microscope. Doubly transduced cells were flow-sorted by the medium intensity in bulk. Use of the EF1a constitutively active promotor is preferred due to its being less susceptible to silencing, therefore, PD-L1 expression can be tightly controlled by the amount of Doxycycline added.

### Reference TMA establishment

A range of PD-L1 expression cell lines were created and embedded into histogel as described previously (20, 21). Briefly, these cells were seeded at 5 x 10^6^ cells per T75 (25 ml of media). The next day cells were treated with increasing amounts of Doxycycline (Santa Cruz Biotechnology, Dallas, Texas, USA) for 24 hours to generate cells with increasing amounts of PD-L1. K562 cells are suspension cells, therefore cells expressing different levels of PD-L1 can be harvested at the same time by centrifugation. Ten percent of the cells were lysed for Western blotting and 90% were subjected histogel embedding. Cells were washed, resuspended, and fixed in 10% formalin (Thermo Fisher Scientific, Burlington, ON, Canada) by incubating on ice for 60 minutes. During this time, cells were counted using the Moxi Z cell counter. Fixed cells were washed and dried cell pellets were resuspended in 65°C molten Histogel (Thermo Fisher, Canada) at approximately 2x10^7^ cells/100µL of Histogel. The gel-embedded cells were solidified at 4°C and overlaid with 70% ethanol until processing into paraffin-embedded blocks. FFPE cell blocks were then constructed into TMAs. These cell lines not only serve as on-slide controls but the PD-L1 expression for each patient is normalized to the cell line TMA to correct for slight differences in staining across multiple patient TMAs.

### Tissue microarray construction and fluorescence immunohistochemistry

TMA construction from archival FFPE tissue has been previously described (4). The PD-L1 inducible cell line TMA was co-mounted to the patient TMA and stained simultaneously. Briefly, heat-induced epitope retrieval was performed by incubating slides in a citrate-based target retrieval solution (Dako, Mississauga, Canada; catalog number S1699), and heating to 121°C for three minutes in a decloaking chamber (Biocare Medical, Concord, CA, USA). Antibodies used were against PD-L1 (Rabbit monoclonal, clone E1L3N, catalog number 13684S, 1:2500, Cell Signaling Technology, Danvers, MA, USA) and pan-cytokeratin (mouse monoclonal, clone AE1/AE3, catalog number M351501-2, 1:100, DAKO, Mississauga, Canada). Isotype control antibodies were used at concentrations matched to those of the respective primary antibodies. IHC was performed on a Dako Autostainer Link 48; antibodies were diluted with SignalStain protein blocking reagent and incubated with the tissue samples at room temperature for 30 minutes. Secondary antibodies were anti-rabbit EnVision+ (K4011, Dako). PD-L1 staining was visualized with TSA-Plus Cy5 signal amplification reagent (Perkin Elmer, Waltham, MA, USA), and nuclei were visualized with 4’,6-diamidino-2-phenylindole (DAPI) (Thermo Fisher Scientific, catalog number D1306). After immunostaining, slides were coverslipped using ProLong Gold anti-fade mounting medium (Thermo Fisher Scientific, catalog number P36934), and stored at 4°C until scanned. For PD-L1, inducible cells and normal tissue specimens (anal epithelium, tonsil, and placenta) were used to optimize the staining conditions, and to determine the minimum effective concentration of primary antibody above which there were no evident differences in staining fidelity or sensitivity.

### Quantitative image analysis

Immunostained slides were digitized using an Aperio ScanScope FL. Identical image acquisition parameters were applied to all stained slides. To determine the mean staining intensity and percentage of PD-L1-positive cells, an algorithm was designed within the HALO image analysis software platform (version 2.0.1145.14, Indica Labs). Briefly, a tumor-specific mask was generated to distinguish the anal cancer cells from surrounding stromal tissue by thresholding the pan-cytokeratin images. Thresholding levels were verified and adjusted, if necessary, by spot-checking a small sample of images to determine an optimal threshold value. All images were then processed using this optimal threshold value and all subsequent image manipulations involved only image information in the masked area. Unusable areas such as folded or necrotic tissue were manually cropped. TMA cores were included in the analysis if 1) at least half of the image was usable and 2) >200 cells per TMA core were present. After review and image analysis validation, data from patients that had usable results were used for subsequent statistical analysis. Tumor PD-L1 positivity score is defined as number of PD-L1 positive tumor cells divided by total number of tumor cells multiplied by 100. This definition is same as pathologist scored TPS.

### Manual scoring of CD8, p16, and p53 expression in anal cancer TMA

Four-micron sections of the anal cancer TMA were subject to IHC analysis. Sections were stained with the Dako Omnis platform using onboard deparaffinization, rehydration, and target retrieval. Incubation time and staining steps were pre-programmed on the Dako Omnis software and all incubation steps were performed at 32°C. Slides were pre-treated using heat-inducted epitope retrieval (HIER) using Dako Omnis high pH EnVision FLEX Target Retrieval Solutions (Agilent Technologies, Carpenteria, California, USA) and stained using prediluted p53 antibody (clone DO-7, Agilent Technologies, Carpenteria, California, USA), 1:24 dilution of p16 antibody (clone E6H4, CINtec, mtm Laboratories, Tuscon, Arizona, US) and prediluted CD8 antibody (clone C8/144B, Agilent Technologies, Carpenteria, California, USA). The Dako EnVision FLEX+ (Agilent Technologies, Carpenteria, California, USA) was used as the visualization system and slides were counterstained using hematoxylin. All manual scoring was performed by a trained pathologist (SL). Interpretation of p53 was based on two patterns 1) wildtype pattern (patchy nuclear staining 1-80%) and 2) mutant pattern including complete absence (with positive internal control), overexpression (strong nuclear staining in >80%) or cytoplasmic expression. Interpretation of p16 was based on three staining patterns 1) absent, 2) normal/heterogeneous and 3) diffuse/block where block expression is described as diffuse staining of all tumor cells in nuclei and/or cytoplasm with strong intensity with virtually no negative tumor cell clusters based on the recommendation from LAST (22). Blocked positive p16 staining has a strong correlation with high-risk HPV. Interpretations of CD8 were based on five density cut off, score of 0 (no CD8+ cells), 1 (low density, 1-2 CD8+ cells per core), 2 (moderate density, 3-15 CD8+ cells per core), 3 (high density, >15 countable CD8+ cells per core), or 4 (extreme density, uncountable CD8+ cells per core). TMA cores with <25% epithelial tumor content were considered uninterpretable.

### Statistical analysis

Results were tabulated and analyzed with SPSS Version 25. Two-tailed Chi-squared or Fisher’s exact tests were used to determine the significance of associations between proportions. The Student’s T-test or ANOVA were used for comparison of means as appropriate. Clinical variables were evaluated for association with survival using Cox proportional hazards model and Kaplan-Meier survival analysis with log-rank test. Variables with significance in univariate analysis (p < 0.05) were included in the Cox multivariate analysis (forward stepwise Wald). PFS is defined as the interval between diagnoses to when the patient recurred/progressed, died (events) or was lost to follow up (censored data point). OS after progression will be calculated from date of diagnoses to death or lost to follow-up.

## Results

### Patient clinicopathologic characteristics

Ninety-nine patients with available biopsy-confirmed anal cancer were included, 63 (64%) had sufficient tumor samples available for full analysis. Patient, tumor, and treatment characteristics are provided in **Table 1**. Of the 63 patients in the analysis, 3 (4.8%) had documented HIV positive status. There were no differences in baseline characteristics between tested and not tested cases (**Supplemental Table 1**). Of the fully analyzed cases, male to female ratio was 1:2.5 with the mean age was 57.3 years (34 to 85 years). The mean tumor size was 4.3 cm (0.7 – 10.0 cm). All patients underwent radical CRT with a median RT dose of 54 Gy with concurrent 5FU/MMC chemotherapy. The mean follow up was 77.5 months (5 – 242 months), the mean PFS was 61.1 months (4 - 226 months) and mean OS was 77.5 (4 - 242 months).

**Table 1:** Patient and tumor characteristics*.

Variable	N (%)
**Age** (mean, year)	57.3 (34 - 85)
**Gender**	
Female Male	71 (72) 28 (28)
**Tumor size** (mean, cm)	4.3 (0.7 – 10.0)
**T Stage**	
1 2 3 4	16 (16) 43 (43) 32 (32) 8 (8)
**N Stage**	
0 1 2 3 X**	75 (76) 7 (7) 10 (10) 5 (5) 2 (2)
**TNM stage**	
I II III IV	14 (14) 57 (58) 27 (27) 1 (1)

*Staging - AJCC 7th Edition.

**N staging not available. Composite TNM stage documented by treating physician.

### P16 (surrogate for HPV) and p53 status

IHC staining for p53 and p16 and quantitative scoring were performed in a subset of patients (65/99, 66%). p53 score was wildtype (WT) in 92.3%. Regarding p16 status, 90.8% of samples tested positive, 1.5% (1 sample) was negative and 7.7% (5 samples) had a patchy staining pattern. A significant correlation between p16 positive and p53 WT status was observed (p<0.0001). Sixty-three cases had results for all biomarkers including p16, p53, CD8 and PD-L1 and were used for subsequent analyses. Fifty-seven (90.5%) were HPV-positive with WT p53, while only one case was HPV-positive with overexpression of p53. One case was HPV-negative p53 WT and 4 cases (6%) were HPV-negative p53 over-expressors. There were no statistically significant associations between p16 (p=0.091) or p53 (p=0.204) expression and tumor PD-L1 expression.

### PD-L1 expression by fluorescence immunohistochemistry

**Figure 1:**
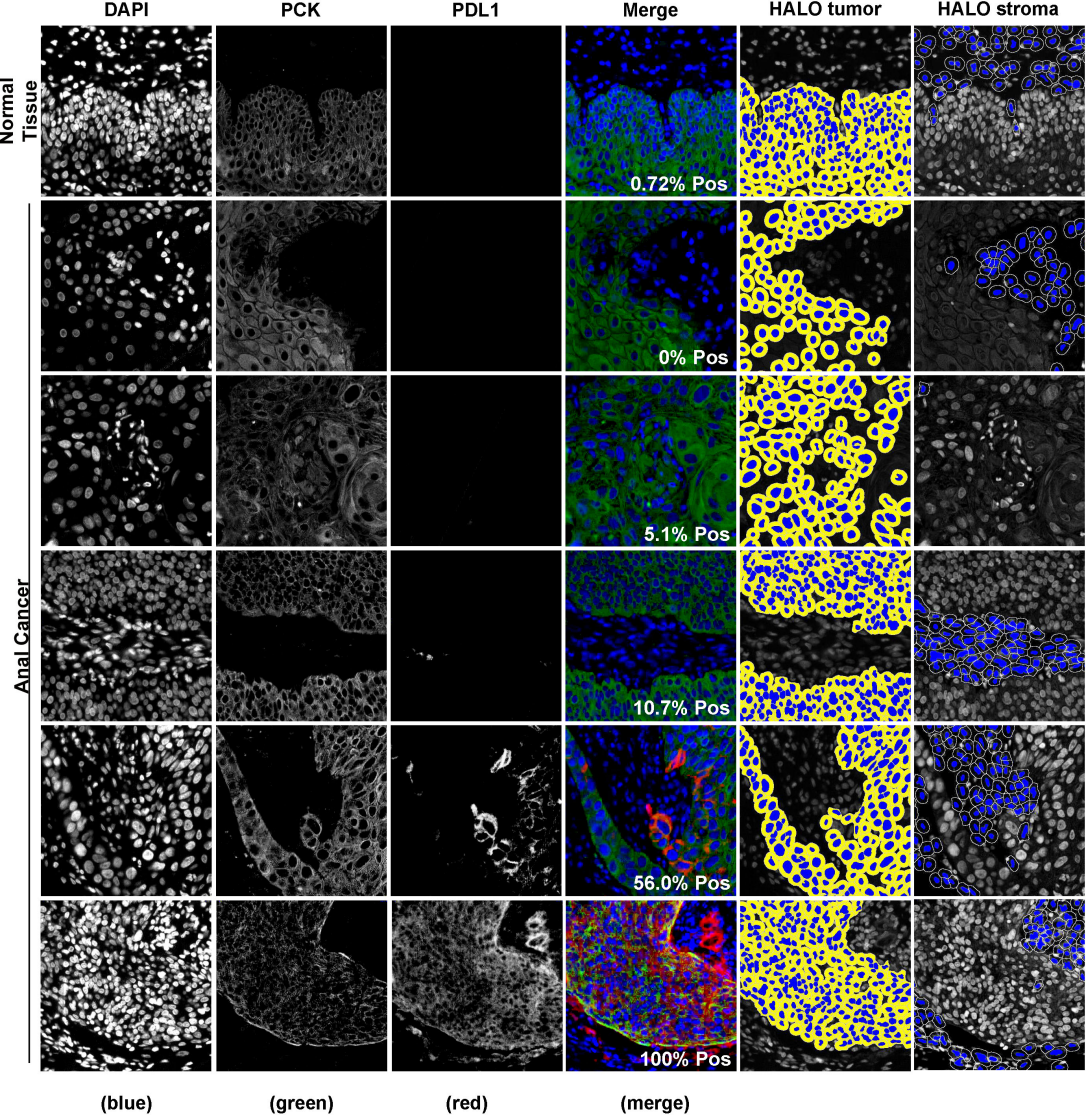
Representative images of fluorescence immunohistochemistry for PD-L1. Samples were visualized using PD-L1 antibody and cytokeratin to identify tumor regions. Digital image analysis using HALO was used to determine the percentage of PD-L1 positivity in the tumor and the tumor inverse mark-up region (HALO tumor and HALO stroma, respectively).

Representative images of PD-L1 staining of normal and anal cancer tissue are in **Figure 1**. The expression patterns of PD-L1 in tumors range from diffuse (uniform throughout the tumor) to sporadic. We used HALO image analysis software to quantify PD-L1 positivity and expression in both stromal and tumor cells. For exploratory analysis, we stratified the cohort into groups based on commonly used cut-points for PD-L1 positivity scores (≥ 0%, ≥ 1%, ≥ 5%, ≥ 10%, ≥ 25% and ≥ 50%). The tumor PD-L1 positivity score and proportion of patient samples are shown in **Table 2**. PD-L1 was negative in 11.1% of tumors. Considering tumor PD-L1 scoring, 50.8% of tumors were positive by the 5% cut-off. For stromal PD-L1 scoring, 47.6% were PD-L1 ≥ 5% and 27% were negative. The mean PD-L1 positivity score in tumor cells was 22.1% and the mean PD-L1 positive in stroma was 17.3%. We used both tumor and stromal PD-L1 positivity score to calculate a simulated combine positive score (CPS); the mean CPS was 38.0 (0 - 217.4). For PD-L1 expression, we used HALO image analysis to quantify PD-L1 expression by pixel intensity as a continuous variable. The expression was normalized to the on-slide reference TMA containing varying levels of PD-L1 expressing cells. The tumor PD-L1 expression ranged from 4.2 to 1108.2 and the stromal PD-L1 expression ranged from 2.7 to 518.4, indicating that PD-L1 was more strongly expressed in tumor cells. In addition, we determined that there is a direct relationship between PD-L1 expression in tumor and stroma (R^2^ = 0.61).

**Table 2:** Number and proportion of patient samples with tumor PD-L1 expression as determined by commonly used cut points, n=63.

PD-L1 +	N	%
>0	56	88.9%
≥1%	45	71.4%
≥5%	32	50.8%
≥10%	25	39.7%
≥25%	18	28.6%
≥50%	11	17.5%

### Correlation of PD-L1 with gender, smoking status, HIV status and other biomarkers

Both tumor PD-L1 positivity score and expression were associated with female gender (T-test p<0.0001 and p=0.002, respectively). Although there was no association between PD-L1 and p16 or p53, there was a significant positive correlation between CD8 expression to tumor and stroma PD-L1 positivity score (ANOVA p=0.0003 and p<0.000, respectively) and expression (ANOVA p<0.000 and p=0.001, respectively). Interestingly, tumor and stroma PD-L1 positivity score and expression were all associated with smoking status (T-test p<0.0001). Notably, there was a statistically significant association between smoking status and tumor PD-L1 expression ≥ 5% (p=0.012). Among patients with tumor PD-L1 <5%, the majority were current smokers (71%); 18% were non-smokers, and 11% were ex-smokers. For patients with tumor PD-L1 ≥ 5% expression, only 27% were current smokers, 20% were non-smokers, and 43% were ex-smokers. The mean tumor PD-L1 score was 44.9% for non-smokers, 31.0% in ex-smokers and 10.3% in current smokers. There was no correlation between HIV status with any of the studied parameters.

### PD-L1 and CD8 expression and survival outcomes

**Figure 2:**
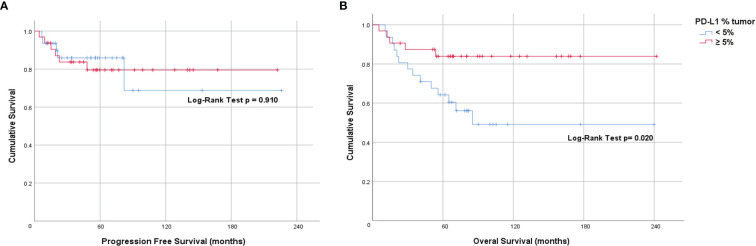
Prognostic value of tumor PD-L1 expression in anal cancer. Kaplan-Meier plots indicating **(A)** Progression-free survival and **(B)** Overall survival of patients stratified by tumor PD-L1 ≥5%.

To determine if PD-L1 positivity score or expression is a prognostic factor for anal cancer, we evaluated its association to PFS and OS using the Kaplan-Meier method *via* PD-L1 mean as well as several cut-points. There was no statistically significant association with PFS when evaluated using the mean PD-L1 positivity score nor mean PD-L1 expression in either tumor or stromal compartment. Additionally, there was no significant correlation of tumor PD-L1 at the 5% cut-point: 10 years PFS: 79% for tumor PD-L1 ≥ 5%, 73% for tumor PD-L1 < 5% (**Figure 2A**). However, when evaluated using the cut-point of tumor PD-L1 ≥ 5%, we found a statistically significant association with OS (Log-rank p=0.020; 10 years OS: 84% for tumor PD-L1 ≥ 5%, 49% for tumor PD-L1 < 5%) (**Figure 2B**). Univariate hazard ratio (UHR) for OS was 0.32 (95% CI 0.11-0.88, p=0.027) (**Table 3**). On multivariate analysis (including tumor PDL1 ≥ 5% and T and N status) only tumor PD-L1 ≥ 5% showed a trend to better OS; HR=0.55 (95% CI 0.12 – 1.00, p=0.052). There is no significant difference in OS when using the common cut-point of tumor PD-L1 ≥ 1% (p=0.499). Additionally, we did not find statistically significant association of simulated CPS with PFS (median CPS log-rank p=0.513; quartile CPS log-rank p=0.664) and OS (median CPS log-rank p=0.128; quartile CPS log-rank p=0.159).

**Table 3:** Univariate analysis for overall survival.

Variable	HR (CI)	P-value
Tumor PD-L1 >5%	0.32 (0.11-0.88)	0.027
Tumor PD-L1 >5% and high CD8	0.62 (0.41-0.93)	0.021
Gender	3.26 (1.59-6.68)	0.001
T-stage	1.76 (1.12-2.78)	0.015
N-stage	1.65 (1.17-2.32)	0.005

**Figure 3:**
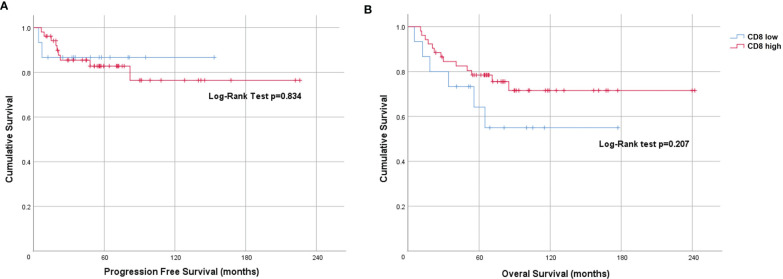
Prognostic value of CD8 expression in anal cancer. Kaplan-Meier plots indicating **(A)** Progression-free survival and **(B)** Overall survival of patients stratified by CD8 score <2 (low) and ≥2 (high).

**Figure 4:**
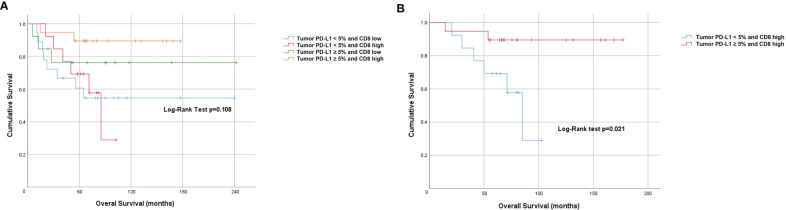
Prognostic value of combined tumor PD-L1 positivity score and CD8 expression. Kaplan-Meier plots indicating **(A)** Overall survival stratified by PD-L1 and CD8 high and low level and **(B)** Overall survival of patients stratified by high CD8 and PD-L1 (<5% and ≥5%).

When dichotomized using a score of < 2 (low) and ≥ 2 or more (high), the Kaplan-Meier curve for CD8 showed a non-statistically significant difference in PFS (Log-rank p=0.834) and OS (Log-rank p=0.207) (**Figure 3**). However, on univariate analysis we found an OS benefit in patients whose tumors expressed both tumor PD-L1 ≥ 5% and CD8 high (score 3 or 4) [HR=0.62 (95% CI 0.41-0.93), p=0.021]. To determine the driver of the survival benefit between PD-L1 and CD8, we evaluated the low and high CD8 within each of the tumor PD-L1 < 5% and the tumor PD-L1 ≥ 5% groups; and we did not find a statistically significant difference in OS between these subsets (**Figure 4A**). However, within the high CD8 group, we observed a significant difference in OS between the tumor PD-L1 ≥ 5% and < 5% (Log-Rank p=0.021) (**Figure 4B**). This suggests the influence on OS benefit is mainly driven by the PD-L1 positivity but may be influenced by CD8 expression. Of note, PFS was not significant when considering both tumor PD-L1 ≥ 5% and CD8 high (Log-rank p=0.764).

## Discussion

PD-L1 has been studied widely as a therapeutic target and a predictive and/or prognostic biomarker in various cancer types. In this study, we investigated the association of PD-L1 and CD8 expression with clinical outcomes in patients with anal cancer treated with radical CRT. We have shown that PD-L1 is expressed in the vast majority of pre-treatment anal cancer specimens, suggesting that anti-PD-1/PD-L1 therapies may potentially be a viable option for these patients. The use of quantitative digital image analysis together with normalization to the on-slide reference cells allowed for a more extensive evaluation of PD-L1 expression levels and positivity scoring than other methods in the literature.

There have been several studies examining the prognostic value of PD-L1 in anal cancer for patients receiving CRT, however the results are conflicting and several analyzed a non-uniform treatment population (13–18, 23) (**Table 4**). It is important to note these previous studies mainly define patients having PD-L1 positivity as any PD-L1 staining in the specimen or at TPS ≥ 1%. This cut-off was established in non-small cell lung cancer and may not be best suited for anal cancer to derive clinical significance. The strength of the quantitative digital image analysis that we have employed in this study is enabling precise intensity measurement and quantification of PD-L1 positive cells in both tumor cells and stroma cells, therefore, allowing the exploration of various cut-points of both compartments in survival and correlation analyses. Furthermore, a strength of our study method is that we incorporated an on-slide reference containing cells of varying PD-L1 expression to normalize pixel intensity across specimens stained on multiple slides to account for slide-to-slide variations and batch-to-batch variations. Lastly, the use of immunofluorescence allows a greater dynamic range and the potential of multiplex additional markers to further explore the role of PD-L1 in the tumor microenvironment.

**Table 4:** Summary of PD-L1 evaluation methods and survival results in previous anal cancer studies.

Study, Year	Histology	Analyzed case number, PD-L1 (n)	Treatment	Staining technique	Antibody clone	Scoring compartment	Cut point	Result for PD-L1 positivity
Balermpas, 2017 (16)	Squamous	150	CRT	DAB	E1L3N	Overall score, not separate intra-epithelial and stromal compartment	Median	DFS NS; p=0.063 (univariate) OS NS; p=0.250 (univariate)
Chamseddin, 2019 (17)	Squamous	16	Heterogeneous (includes surgery and neoadjuvant chemo and neoadjuvant CRT)	DAB	Not specified	Tumor and mononuclear cells	Positive if >1% of tumor (calculated by the sum of stained tumor cells and mononuclear cells over the total number of tumor cells)	OS NS; HR=0.17 (95% CI: 0.03-0.82, p=0.084) (univariate)
Wessely, 2020 (18)	Squamous	33	Heterogeneous (included 20.4% with stage IV disease)	DAB	SP263	Tumor only	TPS various cut points	OS better; HR=0.27 (95% CI 0.10-0.75, p=0.012) (multivariate)
Iseas, 2021 (23)	Squamous	79	CRT; both MMC and cisplatin regimens	DAB	SP263	Tumor and stroma	CPS > 1%	DFS better; HR=0.28 (95% CI 0.11-0.73, p=0.006) (univariate) OS better; HR=0.15 (95% CI 0.03-0.68, p<0.004) (univariate)
Monsrud, 2021 (13)	Squamous	51	Not specified	DAB	22C3	Tumor and stroma	TPS >1% or CPS > 1%	OS worse; TPS HR=3.40 (95% CI 1.18-9.76, p=0.02) OS worse; CPS HR=2.85 (95%CI 1.06-7.67, p=0.04) (multivariate)
Zhao, 2018 (15)	Squamous	26	CRT with or without surgery	DAB	E1L3N	Tumor and tumor infiltrating mononuclear cells	Tumor or immune cells ≥5%	PFS worse; Tumor p=0.038; Stroma p=0.0443 (univariate) OS NS; Tumor p=0.0882; Stroma p=0.1222 (univariate)

DFS, Disease Free Survival.

Similar to KEYNOTE-158 and KEYNOTE-028, our results showed that approximately 71.4% of anal cancer patients have tumor PD-L1 ≥1% (**Table 2**). Our findings establish a new cut-point for anal cancer, given the finding that patients with tumor PD-L1 ≥ 5% have better OS compared to those with tumor PD-L1 < 5%. Interestingly, our results showed no statistical significance with outcome when evaluated using the tumor PD-L1≥ 1% (p=0.499 in OS and p= 0.235 in PFS). This shows the lower cut-point used in various studies may partly explain the controversy in the PD-L1 prognostic effect in anal cancer. Furthermore, the use of a 1% cut-off in the KEYNOTE-028, may partially explain the low overall response rate (ORR) of 17% (95% CI 5.0-37%) for a subgroup of 24 anal SCC patients; 4 patients had a partial response and 10 of 24 patients had stable disease. This may be improved with the 5% cut-off that we found in our study, although further studies with larger numbers of anal cancer patients treated with uniform radical CRT will be required to test this hypothesis.

We found that PD-L1 is associated with CD8 expression. Furthermore, when tumor PD-L1 ≥5% is combined with CD8 high (score 3-4), we see an apparent separation from the remainder of the molecular subtype groupings. This suggests the interplay between PD-L1 and cytotoxic T cells in the tumor microenvironment. Although hypothesis generating, this subset analysis is based on low number of patients and further analysis is required to validate this finding. Although PD-L1 expression on tumor infiltrating immune cells is associated with better prognosis in other cancer types such as breast cancer (24, 25), we did not find a significant association between stromal PD-L1 and OS.

Various studies have examined the association of smoking and PD-L1 status in other cancers, however most focus on patients with lung cancer, and the connection to PD-L1 had never been evaluated in anal cancer (26–29). Interestingly, we found that tumor PD-L1 ≥ 5% was inversely associated with current smoking status. There are higher proportion of patients with tumor PD-L1 ≥ 5% in ex-smokers vs current smokers. This may suggest a change in the patient’s immune response after smoking cessation leading to an increase in PD-L1 expression and more favorable OS. To the best of our knowledge, we are the first to document this in this anal cancer patients, and further evaluation is needed to confirm this finding.

We used p16 as a surrogate marker for high-risk HPV status in our study and determined that over 90% of cases were p16 positive. Roldan Urgoiti et al. have previously demonstrated that p16 is positive in the majority of patients with anal cancer, and there is excellent correlation between p16 expression, HPV status and HPV16 by CISH. Their results support the measurement of p16 as a surrogate marker for HPV infection (4). In our study, there was no statistically significant association between p16 and PD-L1 expression.

We recognize this is a retrospective study with some inherent weaknesses. There may be an inclusion bias as tissue samples were not available for analysis in all of the patients, however we did not see statistically significant differences in the commonly assessed variables between tested and non-tested cohorts. Additionally, validation of this proposed cut-point within clinical trials for anal cancer patients treated with PD-L1 inhibitors will be required to determine if it is more meaningfully associated with response to these agents. Strengths of this study include uniform treatment within two tertiary cancer centers utilizing provincial tumor team guidelines and routine regular follow-up of patients to five years post-treatment.

In summary, using a robust quantitative analysis technique, we have shown that an alternate cut-point for PD-L1 expression (tumor PD-L1 ≥ 5%) is associated with OS in patients with anal cancer treated with curative-intent CRT. PD-L1 expression status using this unique cut-point warrants further validation for prognostication in patients with anal cancer. Future studies are required to determine the benefit of alternative treatment strategies based on PD-L1 status.

## Data availability statement

The raw data supporting the conclusions of this article will be made available by the authors, without undue reservation.

## Ethics statement

The studies involving human participants were reviewed and approved by University of Calgary Research Ethics Board. Written informed consent for participation was not required for this study in accordance with the national legislation and the institutional requirements.

## Author contributions

CD, AC, WJ, SL, EK, PM, RY, EE, AB, MKo, KJ contributed to conception and design of the study. CD, AC, WJ, PM, RY and KJ organized the database. GU performed the statistical analysis. SL, AB and MKo organized the specimen processing and evaluated tumor biopsies to confirm adequacy and sampling locations. AC performed quantitative digital image analysis. SL performed manual scoring for IHC. MK organized specimen collection and performed TMA construction. AC, CD, GU, and SL wrote the first draft of the manuscript. All authors contributed to manuscript revision, read, and approved the submitted version.

## Funding

Funding for this work was provided by the Alberta Cancer Foundation.

## Conflict of interest

The authors declare that the research was conducted in the absence of any commercial or financial relationships that could be construed as a potential conflict of interest.

## Publisher’s note

All claims expressed in this article are solely those of the authors and do not necessarily represent those of their affiliated organizations, or those of the publisher, the editors and the reviewers. Any product that may be evaluated in this article, or claim that may be made by its manufacturer, is not guaranteed or endorsed by the publisher.
