# Supplementary material for: The prognostic impact of PD-L1 and CD8 expression in anal cancer patients treated with chemoradiotherapy
Source: Front Oncol. 2022 Oct 7;12:1000263. doi: 10.3389/fonc.2022.1000263 (PMC9585228; doi:10.3389/fonc.2022.1000263)
Supplement: Supplementary file 1 [file Table_1.docx]

**Supplementary Table 1** - Patient and tumor characteristics* of PD-L1 tested and non-tested cohorts (Total n=99)

| **Variable** | **Without PDL1 results**  n=36 | **With PDL1 results**  n=63 | **P-value** |
| --- | --- | --- | --- |
| **Age** (mean, years) | 58.5 | 57.4 | 0.616 |
| **Gender**  Female  Male | 25  11 | 46  17 | 0.817 |
| **Tumor size** (mean, cm) | 4.4 | 4.3 | 0.709 |
| **T Stage**  1  2  3  4 | 7  17  10  2 | 9  26  22  6 | 0.712 |
| **N Stage**  0  1  2  3  X** | 28  2  3  3  0 | 47  5  7  2  2 | 0.669 |
| **TNM Stage**  I  II  III  IV | 6  20  9  1 | 8  37  18  0 | 0.695 |

*Staging - AJCC 7th Edition

**N staging not available. Composite TNM stage documented by treating physician.
